# Self-Emulsifying Granules and Pellets: Composition and Formation Mechanisms for Instant or Controlled Release

**DOI:** 10.3390/pharmaceutics9040050

**Published:** 2017-11-03

**Authors:** Ioannis Nikolakakis, Ioannis Partheniadis

**Affiliations:** Department of Pharmaceutical Technology, School of Pharmacy, Faculty of Health Sciences, Aristotle University of Thessaloniki, 54124 Thessaloniki, Greece; ioanpart@pharm.auth.gr

**Keywords:** solid SEDDS, controlled release, adsorbents, formation mechanisms, relationships

## Abstract

Many articles have been published in the last two decades demonstrating improvement in the dissolution and absorption of low solubility drugs when formulated into self-emulsifying drug delivery systems (SEDDS). Several such pharmaceutical products have appeared in the market for medium dose (Neoral^®^ for Cyclsoprin A, Kaletra^®^ for Lopinavir and Ritonavir), or low dose medications (Rocaltrol^®^ for Calcitriol and Avodart^®^ for Dutasteride). However, these are in the form of viscous liquids or semisolid presentations, characterized by the disadvantages of high production cost, stability problems and the requirement of large quantities of surfactants. Solid SEDDS (S-SEDDS), as coarse powders, granules or pellets, besides solubility improvement, can be filled easily into capsules or processed into tablets providing a handy dosage form with instant release, which can be further developed into controlled release by mixing with suitable polymers or coating with polymeric films. In this review, the materials used for the preparation of S-SEDDS, their properties and role in the formulations are detailed. Factors affecting the physical characteristics, mechanical properties of S-SEDDS as well as their in vitro release and in vivo absorption are discussed. The mechanisms involved in the formation of instant and sustained release self-emulsifying granules or pellets are elucidated. Relationships are demonstrated between the characteristics of S-SEDDS units (size, shape, mechanical properties, re-emulsification ability, drug migration and drug release) and the properties of the submicron emulsions used as massing liquids, with the aim to further elucidate the formation mechanisms. The influence of the composition of the powdered ingredients forming the granule or pellet on the properties of S-SEDDS is also examined. Examples of formulations of S-SEDDS that have been reported in the literature in the last thirteen years (2004–2017) are presented.

## 1. Introduction

### 1.1. Overview of Self-Emulsifying Drug Delivery Systems (SEDDS)

Emulsion concentrates, described as self-emulsifying drug delivery systems (SEDDS), are composed of drug, oils, surfactants and sometimes co-solvents. They are not themselves emulsions, but on mild agitation in the aqueous environments of the stomach they form easily stable submicron size emulsions. Therefore, SEDDS offer the possibility of solubilization of poorly water soluble drugs. Their presence in the gut, dissolved in fine droplets, avoids the dissolution step of dispersed powder that limits absorption. In this context, the main application of SEDDS is to improve the bioavailability of poorly soluble drugs, which are classified to class II (low solubility, high permeability) of the Biopharmaceutics Classification System (BCS) as Amidon et al. describe [1]. Implementation of the correct SEDDS composition is successful when the dosage form reaches the stomach and the oil/surfactant mixture re-emulsifies spontaneously after mixing with the gastrointestinal (GI) fluids. This mixing is aided by agitation provided by the stomach mobility [2] and it is especially important under fasted state conditions. This is because in this case there is no intestinal content (bile salts and phospholipids) available to assist the emulsification and absorption, with this role being undertaken by the oils and surfactants in the SEDDS. The promising results of earlier studies that showed remarkable absorption improvement of progesterone administered to dogs as SEDDS pellet formulation [3] and of dexibuprofen administered to rats as self-emulsifying powder [4], provided a new perspective and stimulated interest for further studies.

### 1.2. Applications of SEDDS

**Figure 1:**
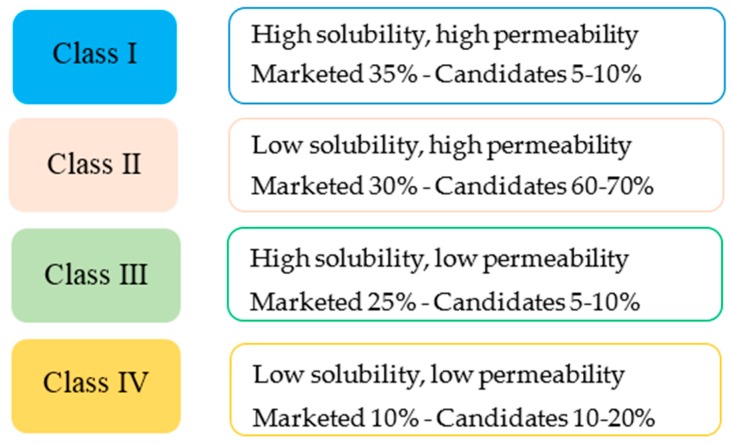
Percentage of marketed drug molecules according to the BCS classification system. Adapted from [13].

According to the Model List of Essential Medicines of the World Health Organization (WHO), from the total approvals of BCS drugs during the period between 2000 and 2011, 41.8% were for Class I drugs (based on both biowaiver and in vivo bioequivalence studies), 20.9% for Class II, 37.3% for Class III, but there were no approvals for Class IV drugs [5]. However, as it can be seen from Figure 1, which presents more recent data up to 2016, Class II now comprises a substantial share (30%) of the marketed products and the largest percentage (60–70%) of the drug molecules under development. This is because the selection of potential therapeutic candidates is based on their ability to bind to cell receptors and since this binding involves hydrophobic interactions, lipophilic drugs are mostly selected for development.

Nevertheless, the improvement of absorption is not limited to BCS class II drugs only. Studies on drug permeability through the GI tract have shown that by the appropriate selection of components, the advantages of SEDDS could be extended to BCS class IV drugs of low solubility and low permeability. Although in the years between 2000 and 2011 approvals for class IV drugs was 0% [5], as shown in Figure 1, in recent years these drugs comprise a significant percentage of 10% of marketed products and 10–20% of drug candidates under development. The application of SEDDS to BCS class IV molecules is based on the ability of SEDDS to improve permeability through the gastrointestinal wall, by influencing physiological and metabolic factors, as well as the function of protein transporters residing in the cell membranes of the intestinal epithelium.

The following mechanisms are implicated for the improvement of permeability:

- Gastric retention time—the oils in the SEDDS can decrease the gastric emptying rate [6].
- Lymphatic transport—the oils in the SEDDS may enhance the lymphatic transportation and the bioavailability of highly lipophilic drugs by promoting their association with chylomicrons in the enterocytes and avoiding hepatic metabolic pathways [7,8].
- Intestinal protein efflux—oils and non-ionic surfactants in SEDDS may reversibly inhibit P-glycoprotein and the multidrug resistance related protein-2 efflux transporters or increase the transcellular permeability [9,10].
- First-pass metabolism—SEDDS may inhibit the action of cytochrome P450 enzymes, which metabolizes drugs in the intestinal wall [10].

Regarding the use of SEDDS to improve the absorption of BCS class I or III drugs, there is meagre information in the literature and a small percentage (5–10%) under development, which is explained due to the minor benefits compared to the other BCS classes. There is one report for the BCS class I drug diazepam [11] formulated into SEDDS showing further improvement of in vitro dissolution and another report for the BCS class III drug gentamicin sulphate (high solubility but low permeability) showing improvement of absorption [12].

### 1.3. Components and Formulation of Liquid/Semisolid SEDDS

Firstly, an account of the oils and surfactants that are used for the preparation of SEDDS will be presented, followed by classification of the different types of formulations.

#### 1.3.1. Oils

In the early SEDDS formulations natural oils such as sesame oil, coconut oil and cotton seed oil were used, which contain mainly low melting point fatty acids (oleic, linoleic, linolenic and ricinoleic). Coconut oil contains additionally small amounts of higher melting temperature fatty acids: caprylic (C8), capric (C10), lauric (C12) and myristic (C14), which are industrially obtained by distillation. Esterification of the medium chain caprylic (C8) and capric (C10) fatty acids yields lipophilic medium-chain triglyceride mixtures (MCT) (glyceryl tricaprylate/caprate) composed of medium chain triglycerides (MCT) C8 (50–80%) and C10 (20–45%) acids, which are extensively used as the oil components in SEDDS (Table 1) [14,15]. This is due to the greater proportion of ester groups in the MCT molecule in comparison to long chain triglycerides, which increase their solvent capacity for drugs and also because they are less amenable to oxidation. Partial hydrolysis of triglycerides yields excipients, which contain various proportions of mono-, di- and triglycerides with greater solubilizing power for less lipophilic drugs [16].

Due to the limited number of oils with high solubilization ability, an increased interest has been recorded recently towards the development of new lipidic carriers of enhanced solubilizing power by esterification of glycerol with short, medium and long chain fatty acids. Depending on the HLB required to form stable emulsions, the esterified products are classified as oils or surfactants in the SEDDS systems [17].

#### 1.3.2. Surfactants

In the formulation of SEDDS, nonionic surfactants have been predominantly used. They have certain advantages over ionic surfactants, such as stability in media with different pH, they do not react with ionic drugs and they are generally compatible with the other formulation ingredients. Esters of sorbitan with hydrocarbon chain length fatty acids known as polysorbates (Span-lipophilic) or after ethoxylation (Tween-hydrophilic) have been extensively used in the formulation of Type II (low HLB < 12), Type III and IV (high HLB > 12) lipid formulations. Furthermore, they can be mixed in different proportions to achieve desirable HLB values and fulfil the requirements of the emulsified oil.

The results of Buyukozturk et al. [18] showed that at high concentrations of surfactants, which have high HLB around 15, they loosen epithelial cell tight junction, whereas surfactants with lower HLB around 10 had toxic effects on the cells. Lipids derived from castor oil (Cremophors) are rich in ricinoleic acid and the corresponding glycerides can be ethoxylated at the hydroxyl group (C12 position of the acid) to increase their hydrophilicity. They are well tolerated after oral administration and they are gaining popularity for use as surfactants in SEDDS, in order to enhance bioavailability of the poorly-soluble drugs (Table 1). Three main products representing this category are: ethoxylated castor oil (Cremophor^®^ EL) and ethoxylated hydrogenated castor oil (Cremophor^®^ RH40 and Cremophor^®^ RH60, BASF Technical Information May 2010 [19]).

In addition, esterification of propylene glycol has been industrially applied for preparing esters with different HLB values, which are used in SEDDS as oily vehicles. Such esters are: propylene glycol monocaprylate type I NF (Capryol™ PGMC), and type II (Capryol™ 90), propylene glycol monolaurate type I EP/NF (Lauroglycol™ FCC) and propylene glycol monolaurate type II EP/NF (Lauroglycol™ 90). As it can be seen in Table 1, they have been employed in the formulation of a number of S-SEDDS products.

Furthermore, the fate of the formulation ingredients in the lumen, where digestion takes place is very important and has been the subject of investigation, among others, of Abdalla and Mader [20] and Vithani et al. [21]. They reported that the replacement of non-digestible surfactants with digestible ones like sucrose esters S-1670 (S-1670) and Span 60 (S-60) eliminated the digestion lag time and reduced the propensity for the formation of colloidal assemblies.

#### 1.3.3. Formulation of SEDDS—Lipid Based Formulation Classification System

The lipid-based formulation classification system (LFCS) proposed by Pouton [2,22] has been used as a general guide. For the formulation of SEDDS. Four types of LFCS were proposed, based on the polarity of the oil/surfactant blends and their ability to keep the drug dispersed in the GI fluids. They effectively ranged from oil only liquids (Type I) to surfactant only semisolids (Type IV), with oil/surfactant blends forming the two intermediate types (Type II and Type III). Type I oil formulations do not disperse in contact with water and they have to be digested by the GI lipases in order to form drug dispersion. This type is suitable for formulating very lipophilic drugs of high LogP. Type II and type III formulations of oil/surfactants blends are able to self-emulsify in contact with water. Type II formulations consist of water-insoluble surfactants with hydrophilic-lipophilic balance, HLB < 12 and produce coarse emulsions in contact with water. On the other hand Type III oil/surfactant formulations consist of water-soluble surfactants, HLB > 12 producing finely dispersed nanoemulsions, which are suitable for the formulation of drugs with LogP between 2 and 4 [23,24].

Type IV formulations consist of water-soluble surfactants and hydrophilic co-solvents. Their advantage is their enhanced solvent power for BCS class II drugs which are hydrophobic but not lipophilic (or which have low to medium lipophilicity), by formation of micellar solutions with solubilized drug upon dilution [22,25]. However, increased proportions of hydrophilic surfactants with polyethylene glycol chains that are needed for Type IV systems cover the oil/water interface and provide steric hindrance to pancreatic lipase action, thus inhibiting digestion and absorption. Also, due to the presence of cosolvent in Type IV LFCS, their solvent capacity decreases after dispersion in aqueous media.

## 2. Solid Self-Emulsifying Drug Delivery Systems (S-SEDDS)

So far, SEDDS have been marketed in the form of liquid or semisolid products. These have the disadvantage that they can only be presented in a liquid-filled capsule dosage form, which although it appears simple to produce, it presents several difficulties. Some of them are the high manufacturing cost due to the low production rate, entrapment of air in the capsule at high speeds of capsule filling and possible incompatibility of SEDDS ingredients with the capsule shell that may reduce the product shelf life [26].

The development of SEDDS into a solid dosage form (S-SEDDS) is another strategy in lipid-based formulation design, which besides solubility improvement, offers further advantages over liquid systems. Such systems involve solidification of the liquid SEDDS mainly into multiple units such as powders, granules and pellets. Consequently, S-SEDDS combine the benefits of liquid SEDDS, e.g., enhanced solubility and bioavailability, with those of solid dosage forms, e.g., easy handling and administration, better patient compliance, high stability and reproducibility, faster and easier production and hence lower production cost). More specifically, they offer the following advantages:

- They reduce the risk of interaction of the ingredients of SEDDS with the capsule shell, thus offering stability improvement due to reduced risk of chemical degradation and microbial growth implying increased product shelf life [26,27].
- They can be administered as immediate or controlled release formulations depending on the choice of the powder excipient, with which the SEDDS liquid is formulated.
- They avoid stringent processing requirements since it is a solid dosage form.
- The dose is presented in precise weight of S-SEDDS powder, granules or pellets filled into a capsule or processed into tablet.
- They are easily transferred and stored, thus improving patient compliance.
- Production cost is considerably less compared to liquid capsule filling since self-emulsifying coarse powders, granules and pellets have excellent flowability, allowing fast and reproducible capsule or die-filling, enabling high production rates.
- Self-emulsifying granules or pellets, in particular, being multiple-unit dosage forms provide therapeutic advantages that are characteristic of these dosage forms. They promote reduction of the variation of the gastric emptying time, smooth passage in the gut and low risk of dose dumping. All these conduce to the minimization of the variability in plasma levels [28].
- More importantly, studies have shown that the release of progesterone in dogs from self-emulsifying pellets was equivalent to administration of the microemulsion liquid [3].

**Figure 2:**
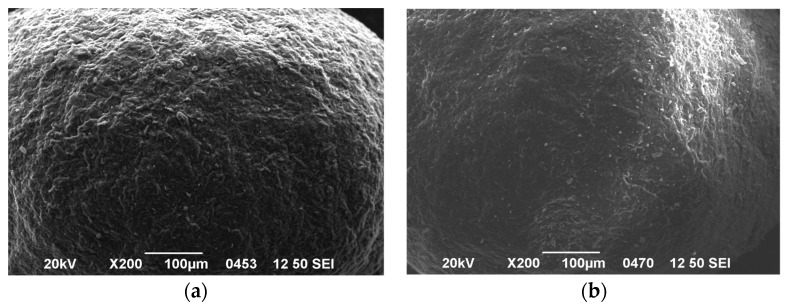
SEM photomicrographs of propranolol pellets: (**a**) without SEDDS and (**b**) prepared with SEDDS as massing liquid with ratio MCT/ELP 6/4 [30].

From the four formulation types of the LFCS classification system, Type I oil based formulations that are intended for the solubilization of very lipophilic drugs can be processed into S-SEDDS only by mixing with adsorbent powders. Granulation and pelletization by extrusion/spheronization cannot be applied since they require aqueous binder liquid [29]. Hot-melt extrusion is a further possibility, though the thermal characteristics of the oily excipients (glass transition or melting temperature) are critical for the feasibility of processing. Type II LFCS producing coarse emulsions and type III producing stable nanoemulsions can be converted into S-SEDDS by mixing with adsorbent powders. However, they are also very suitable for wet granulation and extrusion/spheronization due to the presence of water in the emulsion binder and due to the presence of surfactants with high HLB. The presence of these surfactants assists the spreading of the liquid binder onto the granule or pellet forming powders and the good distribution in the wet mass, resulting in spherical shape and smooth surface (Figure 2).

Type IV surfactant based LFCS, are more suitable for filling into a capsule, in preference to formulating into self-emulsifying granules or pellets, due to the increased viscosity and the tendency to form gel when mixed with water. That is because of their viscous texture that hampers mixing and distribution into the powder components [15,31]. However, the introduction of new water dispersible surfactants of mono-, di- and triglycerides of polyethylene glycol esters of fatty acids [32,33,34,35] and the use of hydrophilic mixtures with lipophilic surfactants have made the conversion of Type IV LFCS into granules and pellets by extrusion/spheronization feasible [36,37,38].

## 3. Components of S-SEDDS

Oils and surfactants, which are added to form SEEDS emulsions that are used as massing liquids, have already been described in the Introduction, Section 1.3. Here, the powder components that form the solid base of granules or pellets are detailed.

### 3.1. Pellet and Granule Forming Powders

#### 3.1.1. Microcrystalline Cellulose (MCC)

Many granulations and nearly all pellet formulations that are produced industrially by extrusion/spheronization contain MCC to different extents as a pelletization agent, in order to ensure successful processing. This is due to the ability of this material to restrain water in its own structure, restricting the separation from the solids during the granulation and extrusion processes, thus yielding a wet mass. This wet mass is characterized by the following: (a) sufficient mechanical strength for enabling the wet mass to retain rod shape after extrusion; (b) certain degree of brittleness required for breaking the rod down to short lengths in the spheronizer; (c) plasticity for enabling the rods to be rolled into spheres by the action of the friction plate in the spheronizer and (d) low adhesiveness required to keep the spherical granules or pellets separated [39].

However, MCC alone as a spheronization aid cannot provide a formulation for all drugs. For example, chemical instability of ranitidine hydrochloride formulated into MCC pellets has been reported [40]. Another important issue related to the use of MCC in formulations, where it is in suspension or wet processed together with certain drugs, is the preferential adsorption of the drugs onto MCC [41]. The uncontrollable adsorption of a drug onto solid dosage form excipients may influence its dissolution characteristics, analytical testing and bioavailability. This is particularly important for drugs which are normally used in low doses, such as ketotifen fumarate which was found to adsorb onto MCC, croscarmellose sodium and pregelatinized starch [42]. Studies of the adsorption of various drugs onto MCC suspended in aqueous solutions showed that adsorption of four phenothiazine derivatives was considerable and that of acrinol was quite large [43]. Other studies have reported adsorption of famotidine [44]. Furthermore, the disintegration of pellets based on MCC varies, depending on the type of MCC and on the wetting and drying conditions [45]. In addition, the drug release is not slow enough to produce an extended release dosage form and for that reason the application of polymeric coatings is required [46].

#### 3.1.2. Adsorbents—Potential Alternatives to MCC

In a strict sense, adsorbent powders (adsorbents or otherwise ‘carriers’) are chemically inert substances that are able to physically adsorb the liquid or semisolid SEDDS after mixing in order to produce S-SEDDS as self-emulsifying powder. Certain silicon oxides and silicate salts fulfil the requirements both as adsorbents and as granule or pellet forming agents. Pharmaceutical diluents like MCC, lactose, cellulose and starch derivatives, although they have adsorption ability, they are not considered as such carriers. That is because their adsorptivity is generally low and their role in the formulation is different.

Since the solubility of the drug in the liquid SEDDS is generally limited and incorporation of SEDDS into solid carriers causes further decrease of the loadable drug levels in the final granules or pellets, developability may be a problem. For this reason, it is important to select adsorbents with high adsorption ability, so as to take up high volumes of liquid SEDDS with dissolved drug and yield acceptable drug loadings in the final product. Adsorbents, such as colloidal silicon dioxide (CSD) and silicate salts that are used as pellet base are able to uptake large amounts of SEDDS emulsions. Due to the adsorptive ability, they are able to retain the SEEDS within the wet mass during wet granulation and during extrusion and spheronization, and also in the final dry granule or pellet after removal of water. This finally leads to a greater content of drug in the final dry pellet.

From Table 1 it can be seen that different grades of silica (silicon dioxide), colloidal silicon dioxide and magnesium aluminosilicate, which are pharmaceutically acceptable excipients with high water retention capacity, have been used as adsorbents in the preparation of the solid dosage forms. One case where mannitol was used has also been reported [47]. Colloidal silicon dioxide (CSD) or fumed silica (NF/USP), commercially available as Aerosil^®^ (Evonic, Germany), is offered in various grades differing in their specific surface area from about 200 m^2^/g to 300 m^2^/g, in their hydrophilicity and also in their packing ability and mixing behaviour.

Neusilin^®^ range comprises another type of adsorbents (USP/NF), which are amorphous solids chemically based on magnesium aluminometasilicate and that unlike CSDs, do not form gel in aqueous environments [48]. They are also offered in various grades, differing in their specific surface area from about 100 to 300 m^2^/g, in their particle size, packing ability and mixing behavior, and also in their pH, so that chemical compatibility with acidic or basic drugs is achieved by proper selection.

Silicon dioxide products with high specific surface area and internal porosity and also with high adsorptivity to both hydrophilic and hydrophobic materials, but with greater particle size of about 5 μm, are also available (SYLOID^®^ 244FP) [49]. The larger particle size improves handling and processing by reducing dust and facilitating incorporation into the formulation [45]. It has also been reported the use of further grades of silica derivatives: calcium silicate (25 μm), magnesium aluminum silicate (5 and 80 μm), and silicon dioxide (3.6, 20, and 300 μm) are available in granular form, which is easier to handle and process [50].

Magnesium aluminometasilicate has also been used as a bifunctional excipient pellet aid and as adsorbent [33,35,51,52,53,54]. Milovic et al. [52] studied the effect of different adsorbents on the release of carbamazepine (CBZ) from S-SEDDS and found decrease in the release rate with increasing specific surface area of magnesium aluminometasilicate adsorbents, caused by entrapment of liquid self-microemulsifying drug delivery systems (SMEDDS) inside the pores of the adsorbent. Chavan et al. [38] also studied the impact of the properties of different silicon dioxide adsorbents on drug release of celecoxib and found considerable differences which were ascribed to the different surface area, the porosity and the hydrophobicity–hydrophilicity.

### 3.2. Controlled Release Agents

Besides improvement of drug solubility by formulating into S-SEDDS, it would be desirable to combine this solubility improvement with prolonged release by adding suitable agents. In this context, it is noticeable that in addition to their adsorption power and pellet forming ability, certain adsorbents may prolong drug release. In the case of CSD this is due to gel formation in contact with water. A three-dimensional (3D) network is formed, resulting in delayed drug release by diffusion through the CSD gel. The rate of diffusion depends on the gel viscosity or on the type of CSD and its proportion in the formulation. Patil et al. [55] formulated a sustained release S-SEDDS of ketoprofen as the drug, Captex 200 as the oil, Tween 80 as the surfactant and Capmul MCM as co-surfactant, and CSD as the solidifying excipient. CSD increased significantly the viscosity of liquid crystal in the self-emulsification process which in turn increased the average droplet size of resultant emulsion and slowed down the drug diffusion and the release. Furthermore, from Table 1 it can be seen that CSD has been used for the preparation of sustained or controlled release pellets of tetrahydrocurcumin [56] and ibuprofen [57].

Cellulose ethers offer another possibility for modifying drug release from S-SEDDS. Among them, hydroxypropyl methylcellulose (HPMC) is an established water-soluble, non-ionic cellulose ether that gels in water forming swellable hydrophilic matrices. It is stable over the pH range 3.0–11.0 and enzyme resistant. It is used in order to provide the controlled release of a drug, which diffuses through the hydrophilic gel structure [58,59]. Several workers have used combinations of MCC with HPMC as the pellet forming powders of self-emulsifying pellets, so as to achieve controlled release of drugs (Table 1). For example, Zhang et al. [60] developed self-emulsifying sustained release pellets of puerarin for oral bioavailability enhancement, and Tao et al. [61] prepared sirolimus (SRL) self-emulsifying powder by mixing SEDDS with low viscosity HPMC grade as tabletting and sustained release aid.

### 3.3. Crystallization Inhibitors and Other Additives

After the formulation reaches the stomach, it emulsifies spontaneously in contact with the GI fluids. Under the action of pancreatic lipase, the oils in the formulation undergo de-esterification into fatty acids and partial glycerides, which affects the emulsion stability. It is, therefore, important to ensure that the poorly soluble drugs remain solubilized in the oil/surfactant phase, without crystallization before facing the enterocytes. One of the first literature reports in this topic was the work by Gao et al. [62] and by Wei et al. [63] who developed supersaturable S-SEDDS employing hydroxypropyl methylcellulose (HPMC-E5LV) and HPMC respectively as precipitation inhibitors, in a conventional SEDDS formulation.

Besides HPMC, other novel polymers have also been tried as crystallization inhibitors. Song et al. [64] used Soluplus^®^ as precipitation inhibitor in celecoxib S-SEDDS formulation (Capryol 90 as the oil, Tween 20 as the surfactant and tetraglycol as co-surfactant). The S-SEDDS formulations were tested for drug dissolution in gastric fluid. They found that the solubility of celecoxib in S-SEDDS without Soluplus^®^ increased in the initial period of 5 min, but decreased after that period of time. On the contrary, the solubility in S-SEDDS containing Soluplus^®^ was concentration-dependent, showing greatest dissolution of approximately 90% with delayed drug crystallization.

Besides pellet forming agents, adsorbents and crystallization inhibitors described above, other components in powder form—like antioxidants and disintegrants—may have to be added into S-SEDDS formulations for stability purposes. Tao et al. [61] optimized S-SEDDS formulations by adding 0.20% of citric acid to increase the stability of SRL under high temperature (40 ± 2 °C), humidity (relative humidity 90 ± 5%) and strong light irradiation (4500 ± 500 lx).

## 4. Formation Mechanisms

### 4.1. Effect of Drug Incorporation on the Characteristics of SEDDS in Water Emulsions

Certain drugs have been found to affect the properties of SEDDS in which they are solubilized. Pouton [92] studied the effect of adding a range of concentrations of benzoic acid in a self-emulsifying system (30% *w*/*w* Tween 85/70 *w*/*w* Miglyol 812) and compared their emulsification efficiency in either water of 0.1M HCl. Differences were correlated with modifications to the phase diagram related to the ability for liquid crystal formation. Sznitowska et al. [93] found that interaction of drugs with submicron emulsions is complex in nature and that it was difficult to predict changes in the physical stability of the system from physicochemical properties of a drug, such as e.g., lipophilicity or ionization. If destabilization occurred it was maximal at saturated drug concentrations and the presence of undissolved drug did not influence the short-term stability of the system.

Patil et al. [94] found an inverse relationship between droplet size of the formulations containing structural analogues of ibuprofen and their LogP values. Microstructural analysis of intermediate hydrated regimes of the prepared samples showed formation of local lamellar structure. Structural analogues of ibuprofen of different LogP significantly altered the microstructure of lamellae, which was well correlated with the droplet size of the final formulations. In vitro drug release study showed an increase in dissolution rate of lipophilic drugs, when formulated into SEDDS.

**Figure 3:**
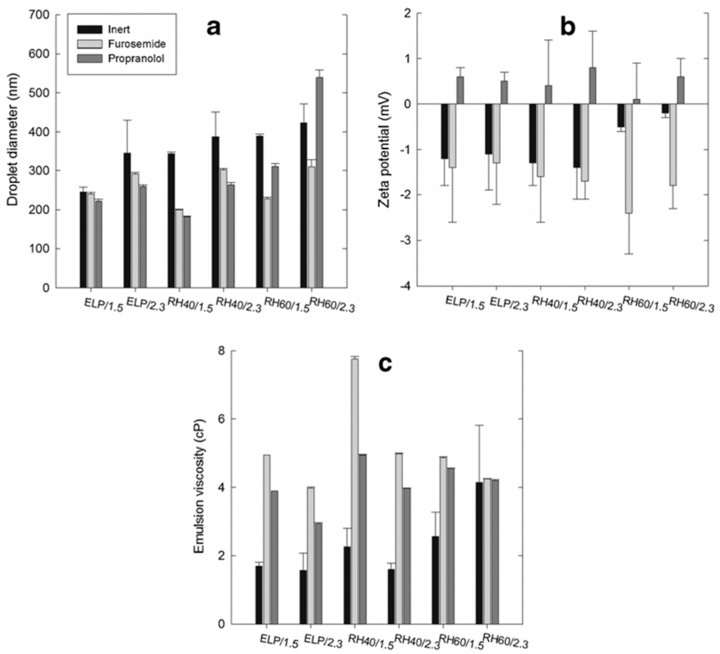
Droplet diameter (**a**); zeta potential (**b**) and viscosity (**c**) of nanoemulsions of MCT/Cremophor 6:4 before and after addition of furosemide or propranolol [mean, (SD), *n* = 3] (reprinted from [31] with permission (Springer 2015)).

Further studies [31] showed that solubilization of drugs to SEDDS III systems not only changed the particle size of the inert emulsions, but also changed spectacularly the charge of the surface of the emulsion droplets (Figure 3). In addition, the viscosity of emulsions was affected by the presence of the drug in the SEDDS, which was attributed to its distribution in the oil/water interface and to the consequent alterations in the surrounding the droplet hydrated layer and shearing resistance.

### 4.2. Self-Emulsifying Powders and Granules

In general, the preparation of self-emulsifying granules and pellets resembles the process of conventional granulation and extrusion/spheronization. The main difference is in the use of the SEDDS in water emulsion as massing liquid and binder, instead of an aqueous solution of a conventional polymeric binder such as polyvinyl pyrrolidone. The use of SEDD emulsions is expected to improve spreading and distribution of the SEDDS in the powder components, thus improving sphericity and size uniformity, although some deteriorating effect on the mechanical strength cannot be avoided [24,31].

Agarwal et al. [50] used a powder rheometer to study the rheology of SEDDS with adsorbents during granulation, in order to understand the effect of SEDDS on the powder flow and to characterize the wet granulation process. They correlated this effect of SEDDS with the stepwise or continuous growing of granules. The same workers also reported that adsorbents exhibited an initial lag phase, during which there was no change in flow which was attributed to their porous nature. They concluded that particle size, specific surface area, type and amount of adsorbent are important factors that determine the flow of self-emulsifying powder.

Cavinato et al. [95] studied the mechanism of granule formation and compared the binder performance of pure water and of SEDDS (Lauroglycol 90, Cremophor EL, and Transcutol) in water emulsion. They found that more spherical granules with narrow size distribution were obtained using SEDDS-emulsions as a binder and when the massing phase is performed at low impeller speed. Furthermore, increasing massing time increased the granule strength, when water was the binder, but did not affect the strength of granules prepared with SEDDS-emulsion. Two types of granule growth were distinguished: (a) one for granules prepared with water as a binder, which is characterized by quick growth, yielding brittle fragmenting granules and (b) the other for granules prepared with high viscosity SEDDS-emulsion, which is characterized by slow growth, yielding higher shear resistance and low fragmentation propensity granules with more spherical shape.

### 4.3. Instant Release Self-Emulsifying Pellets

Newton et al. [29], in one of the first publications on S-SEDDS, found that water was an essential element of the formulations and that the maximum quantity of oil/surfactant that could be incorporated was 42% of the dry pellet weight. Furthermore, the same group of researchers [96] applied a ram extruder as a method of characterizing the wet powder masses, which were formed from different ratios of self-emulsifier (mixed mono- and diglycerides of caprylic and capric acids) and water added to equal parts of MCC. They identified three regions of behaviour of the systems, which were all significantly different from the systems containing only water and MCC. At low self-emulsifier contents (1.5–23%) the masses increased their resistance to shear and elongational flow and had lower elasticity, whereas at high self-emulsifier contents (69%, 80% and 92%) the systems showed less resistance to shear and flow, but considerably higher elasticity and at the mid 46% content the behavior was also intermediate. At 46% self-emulsifier, changes in the ratio of the emulsion (formed by adding water to the self-emulsifier) to MCC, resulted in a change in the values of the rheological parameters, but not in the rheological behaviour.

Matsaridou et al. [24] used different Cremophor grades to study the effect of surfactant HLB on the preparation, characteristics and mechanical properties of self-emulsifying pellets. They found that water requirements for pelletization increased linearly with increasing HLB. Incorporation of higher HLB surfactants enhanced H-bonding, and resulted in faster and more extensive disintegration of MCC as fibrils. Moreover, the less hydrophilic Cremophor ELP grade with a double bond in the fatty acid showed weaker H-bonding, but greater microemulsion reconstitution.

### 4.4. Controlled Release Self-Emulsifying Pellets

Due to their spherical shape, smooth surface and narrow size distribution, self-emulsifying pellets are particularly suitable for sustained release formulations. These formulations are prepared either by application of a sustained release coating from a polymeric dispersion or solution onto the drug containing pellets, or by employing mixtures of pellet forming powder excipients, mainly microcrystalline cellulose (MCC) with sustain release agents to form a sustained release matrix. These excipients are usually gel-forming hydrophilic polymers, such as cellulose ethers (e.g., hydroxypropyl-methylcellulose, HPMC), methacrylic acid based polymers (Carbopol), natural hydrophilic colloids (e.g., chitosan combinations with oppositely charged natural polymers such as sodium alginate), or colloidal silicon dioxide and less frequently esters of long carbon chain fatty acids. In Table 1 are presented several cases of controlled release S-SEDDS reported in the literature.

High efficiency of the control release excipients is very important since their incorporation in the SEDDS reduces further the drug content in the final dry product, and hence developability. As an alternative option to classical excipients, control release agents with dual functionality, i.e. high adsorption ability for SEDDS combined with the ability to control drug release are required [97]. This is further discussed later in the chapter. Nevertheless, if a sustained release S-SEDDS formulation of matrix type is not possible, e.g., due to the high drug content, control release can still be achieved by application of suitable polymeric coatings from organic solutions or aqueous dispersions as said above.

**Figure 4:**
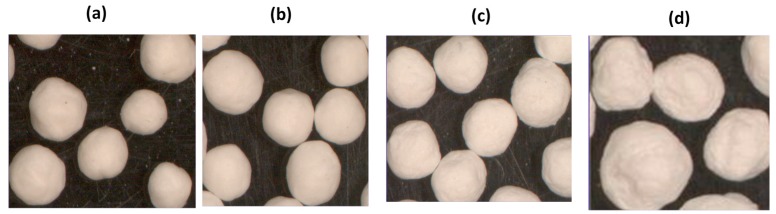
Stereoscope microphotographs of pellets with CSD/MCC ratios: (**a**) 0/10; (**b**) 3/7; (**c**) 7/3 and (**d**) 10/0 [57].

Experiments with CSD in mixtures with MCC using SEDDS added as emulsions-binders in extrusion/spheronization process showed that good quality pellets can be formed [57,97]. Therefore, CSD serves a multi-purpose; as a pellet forming powder, as a strong adsorbent and as gelling agent providing controlled release [34,67,68,98]. These properties of CSD are demonstrated in Table 2 and in Figure 4. Table 2 shows data for emulsion consumption, pellet diameter and shape factors expressed as aspect ratio and shape factor e_R_ (the more spherical the pellets the higher the value of e_R_) [99] for ibuprofen pellet batches prepared with medium chain triglycerides and glyceryl polyethylene glycol oxystearate (Cremophor RH 40). It is noticed that the emulsion consumption, and hence the drug content in the final dry pellets increase greatly with CSD content, becoming more than double at CSD/MCC ratio 7/3 (Table 2). From Table 2 it is also noticed that the median pellet diameter increases with CSD, which is confirmed by the photomicrographs of the pellets shown in Figure 4. This is explained due to the increased pellet mass caused by the greater emulsion consumption.

However, considering the morphology and most importantly the mechanical properties of the pellets that are produced using adsorbents only as pellet forming solids, these may not satisfy the requirements for further processing, necessitating addition of some MCC. Smooth and spherical pellets (AR ≤ 1.13 and e_R_ ≥ 0.419) can be produced up to CDS/MCC ratio 7/3 (Table 2 and Figure 4c), whereas pellets prepared only with CSD (Figure 4d) have a rough surface and greater deviation of the shape parameters from the unity. More importantly, % friability of pellets prepared with CSD/MCC ratios above 7/3 increases considerably, reaching unacceptable friability values >3% [57].

This indicates that although CSD with SEDDS massing emulsion does form pellets on its own, these pellets may have poor size and shape characteristics and not enough strength for further processing.

**Figure 5:**
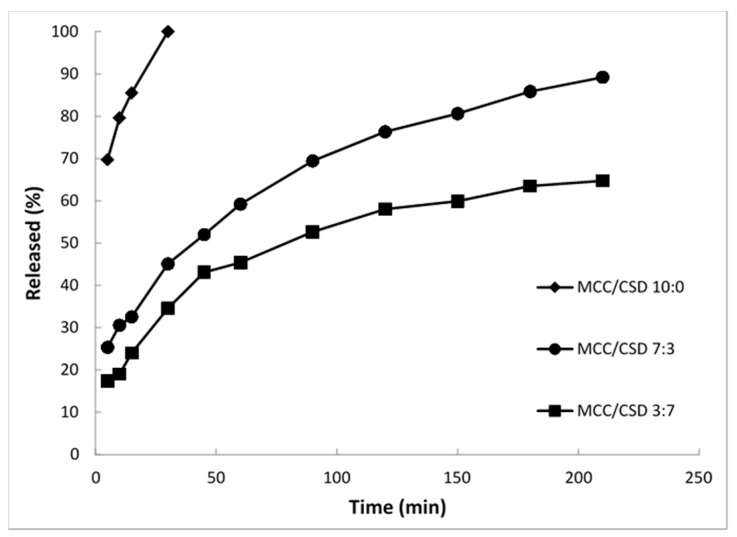
Release of ibuprofen from self-emulsifying pellets with different MCC/CSD ratios in deionized water (pH = 5.9, sd < 8%) [57].

Figure 5 shows the drug release vs. time profiles of ibuprofen from self-emulsifying pellets with different MCC/CSD ratios in deionized water (pH = 5.9). It can be seen that the curves of the different self-emulsifying pellets fall into three different locations: (a) one with pellets prepared without CSD showing instant release; (b) a second group with pellets of CSD/MCC ratio 3/7 showing slow release, which is completed within 4 h and (c) a third group with CSD/MCC ratio 7/3 showing much slower release with about 70% completed in 4 h. Therefore it appears that CSD in combination with MCC provides pellet formulation with sustained drug release.

## 5. Relationships between the Characteristics of the Starting Massing Emulsions and the Properties of the S-SEDDS Pellets

Since in the context of this review administration of SEDDS is not considered in their liquid form but only after transformation into solid self-emulsifying units, it would be desirable to be able to predict the performance of the final dry product. This prediction could be made from the properties of the original SEDDS in water emulsion which is incorporated into the product as massing liquid/binder, from the properties of the individual powder components, and their proportion in the powder forming mixture and from the SEDDS/solids ratio. All these could be related to the properties of the final S-SEDDS pellets.

### 5.1. Droplet Size, Zeta Potential and Viscosity

Droplet size is an important characteristic of the reconstituted emulsion from the S-SEDDS product because it affects the rate of drug release and the absorption [100,101]. From the studies reported in the literature it appears that after reconstitution of S-SEDDS in water the measured droplet size is generally greater than that of the original massing emulsion. However, it is still in the nanosize range below 300 nm [37,68,102,103].

In a recent work [31], possible relations between the characteristics of original massing nanosize range emulsion with the characteristics of the corresponding reconstituted self-emulsifying pellets were examined. The droplet size of the reconstituted emulsions depended mainly on the solubility of the drug in the SEDDS and on the stability of the original nanosize emulsions. Pellets prepared with SEDDS composed of medium chain triglycerides and different Cremophors gave after reconstitution emulsions with smaller droplet size when propranolol (LogP = 3.48) was solubilized in the SEDDS compared with pellets of the same oil/surfactant/solids but with furosemide (LogP = 2.03) as the solubilized in the SEDDS drug. This was attributed to the greater solubility of the more lipophilic propranolol in the SEEDS, remaining dissolved in the droplet during pelletization and drying, resulting in easier and more homogenous reconstitution with smaller droplet size. The increase of the average hydrodynamic diameter in the reconstituted emulsions compared to the original, was by about a factor of 2 but still in the nanosize range.

In the same work [31], the effect of the stability of the original massing emulsion on the droplet size of the reconstituted emulsions was examined using homologous Cremophor surfactants of different HLBs. The higher HLB 15.7, Cremophor RH 60, produced less stable emulsions which was reflected in a marked increase of droplet size and higher polydispersity indices (PDI) of the reconstituted emulsions compared to Cremophor RH 40 with lower HLB 14.3. The increase in the droplet size of the reconstituted emulsions was attributed to the insufficient concentration of surfactant in the droplet/water interface during re-emulsification, due to the delayed availability of the SEDDS components to the reconstituting liquid (water) in the case of less stable emulsions. In general, the more stable the original emulsion is, the more stable will be the reconstituted emulsion and with smaller droplet size. In addition, the zeta potential of the reconstituted emulsions changed considerably towards negative values, which was ascribed to the effect of dilution on the composition of droplet/water interface, and consequently in the diffuse electrical layer around the droplets.

Further examinations between the characteristics of the original massing emulsions used for extrusion/spheronization and the physical properties of the self-emulsifying pellets showed that linear relationships existed between the viscosity of the massing nanosize emulsion and the shape parameters (aspect ratio and roundness) of the pellets [31]. The increased sphericity with decreasing viscosity was attributed to the more even spreading of emulsions on the pellet forming powder resulting in more homogeneous wet mass with improved distribution of the SEDDS in the pellets.

**Figure 6:**
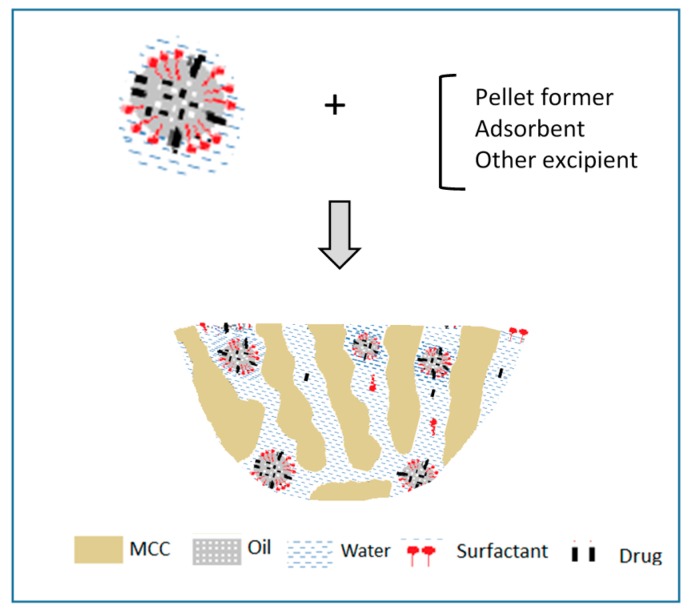
Schematic representation of drug, surfactant and oil in MCC pellets (reprinted from [31] with permission. Springer 2015).

In the same work [31], the migration of furosemide and propranolol drug towards the surface of self-emulsifying pellets during extrusion/spheronization/drying was studied (Figure 6). Migration was greater for the more fluid SEDDS prepared with Cremophor EL (liquid) compared to Cremophor RH (semisolid) and also greater for the higher (7:3) oil/surfactant ratio.

In addition to the viscosity (*η*) of the SEDDS emulsion, it was found that drug migration depended on the drug solubility (*S*) in the SEDDS. Migration (M %) was found to decrease exponentially with the product [*η*·*S*], following a simple exponential equation (*r*^2^ = 0.856):

M % = 98.1 exp − 0.016[*η*·*S*] (1)

### 5.2. Rate of Re-Emulsification of Emulsion from the S-SEDDS and Drug Release

Since the drug is transferred from the S-SEDDS units into the dissolution medium solubilized in the oil/surfactant droplets, the rate and extend of release is expected to be controlled by the rate of re-emulsification and completeness of reconstitution. The results of the work of Matsaridou et al. [24] showed that re-emulsification rate is mainly affected by the oil/surfactant ratio in the SEDDS carrier or surfactant content. At lower oil/surfactant ratios or increased surfactant content the rate is slower, because of the gel formation, impeding penetration of water into the viscous layer and re-emulsification [104].

The contribution of the rate and extent of re-emulsification to drug release was confirmed in another work [71] using self-emulsifying pellets prepared with medium chain triglycerides/Cremophors as self-emulsifying system and propranolol, furosemide as drugs of different lipophilicity (LogP 3.48 and 2.03 respectively). Re-emulsification ability was expressed as the area between the curve of light transmittance T% (λ = 850 nm), decreasing with time due to the developing turbidity of the reforming emulsion and the line parallel to the horizontal time axis drawn at T = 100.0%. A significant linear relationship was found for both drugs between the reconstitution rate constant and the release rate constant of the burst phase of drug release, indicating strong dependence of the last on re-emulsification and existence of excess SEDDS on the pellet surface.

Drug release was expressed by a biexponential first order equation, which takes into account two different release mechanisms:

100 − M_t_ =*A* exp(−*k_a_t*) + *B* exp(−*k_b_t*) (2)

where *A* and *B* are parameters representing the % released achieved by each of the two mechanisms and *k_a_* and *k_b_* are the corresponding release rate constants having dimensions of inverse time. Additionally, piecewise linear regression was applied to determine the time (τ) of the change of the operation and the duration of each release mechanism. The release constant *k_a_* of the burst phase was higher, while the duration of this phase (τ) was shorter for the high oil/surfactant ratio or low surfactant content pellets, which can be attributed to the greater accumulation of emulsion on the pellet surface migrating towards the surface of the pellets during drying.

**Figure 7:**
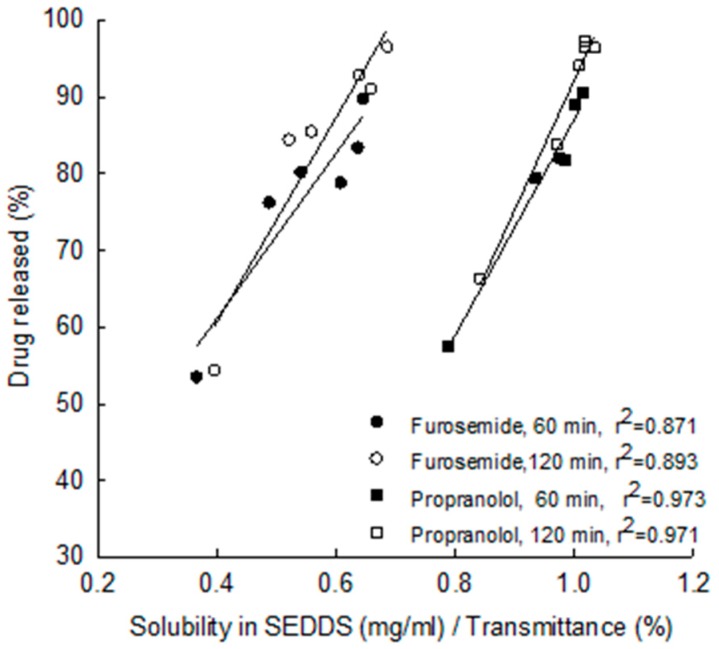
Plots of drug released (%) after 60 min (solid symbols) and 120 min (open symbols) vs. the ratio of the solubility in the oil/surfactant mixture over transmittance (reprinted from [71] with permission. Elsevier 2015).

From the same work [71] it was also found that the release depended strongly on the drug solubility in the oil/surfactant mixture. Linear relationship was obtained between the drug released (%) after 2 h and the ratio of drug solubility in SEEDS over transmittance (r^2^ = 0.893 and r^2^ = 0.971 for furosemide and propranolol respectively) (Figure 7).

### 5.3. Mechanical Strength

Although improvement of drug absorption and control of drug release is the main objective of the solid self-emulsifying drug delivery systems (S-SEDDS), these should not be accomplished at the expense of mechanical strength. The reason is that self-emulsifying powders, granules and pellets comprise multi-unit drug delivery systems, which are not administered as such. Filling into capsules or into the dies of tabletting machines for compression is necessary and this requires nearly spherical shape and narrow size distribution of the multiple units, in order to ensure good particulate flow, enabling successful processing at high speed machines [105].

Furthermore, if the S-SEDDS multiple units are to be coated for controlled release besides narrow size distribution and sphericity, they should also possess low friability to withstand frictional forces against each other. This may be an issue when formulations with low MCC contents are prepared and extra binders such as PVP may have to be added to the SEDDS, in order to enhance the strength of the resulting pellets prepared by extrusion/spheronization. Also, if the S-SEDDS units are to be compressed into tablets, they should have mechanical strength to be able to withstand the compression forces without breaking.

## 6. Examples of S-SEDDS Formulations—Instant Release and Controlled Release

In general, S-SEDDS require drugs of low solubility in water but relatively high intrinsic lipophilicity since they have to be dissolved in a small amount of oil. Additionally, high chemical stability in the oil phase and low crystallization tendency (low ratio of melting over glass transition temperature) of the drug are desirable, so as to remain in solution without crystallizing when it is in contact with the GI fluids in the stomach. In order to gain a better understanding of the reasons for successful formulation, Thi et al. [23] examined the formulation ability of poorly water-soluble drugs in SEDDS composed of different, oils and surfactants with either high HLB > 12 or low HLB < 10. They suggested an optimal LogP of the drugs between 2 and 4 for successful formulation into Type III lipidic systems producing stable nanoemulsions over oil/surfactant ratios from 1.5 to 3.1 [24,31].

Table 1 presents examples of S-SEDDS formulations that have been developed and published during the last thirteen years (2004–2017). Most of the formulations contain drugs with LogP between 2 and 4 [23]. However, a considerable number of studies report S-SEDDS with drugs of quite low lipophilicity with LogP < 2 [46,50,51], which are formulated using Type III LFCS, and a considerable greater number report S-SEDDS with drugs of quite high lipophilicity with LogP > 4 [32,33,34,37,61,65,66,67], which are formulated using surfactant based Type IV LFCS. Most of the S-SEDDS cases presented in Table 1 pertain to solubility improvement of BCS class II drugs (67.6%) and fewer cases for the improvement of both solubility and permeability of the class IV drugs (14.7%). There is also one case of BCS class III drug, gentamicin sulphate, formulated as SEDDS with Labrasol (PEG-8 Caprylic/Capric Glycerides, HLB 12) and Tween 80 and further converted into solid by kneading with silicates [12], which reported high plasma levels in dogs after oral administration as an enteric capsule. Moreover, improvement of the in vitro dissolution of BCS class I drug has also been reported for diazepam formulated into SEDDS with C18 with mono- and diglycerides (Cithrol GMS) and into S-SEDDS by extrusion/spheronization with microcrystalline cellulose (MCC) [11].

Mainly three S-SEDDS forms have been studied and detailed in Table 1: self-emulsifying coarse powders prepared by simply mixing SEDDS with adsorbent powder followed by sifting through coarse sieves (apertures between 500 and 700 microns), self-emulsifying granules prepared by mixing and kneading in a granulating equipment, and self-emulsifying pellets prepared by wet mixing and kneading followed by extrusion and spheronization. In addition, the oil and surfactant components that were used to formulate the S-SEDDS and the powdered solids that were used to convert them into S-SEDDS are given in Table 1 together with the method of evaluation and achievement e.g., in vitro and in vivo improvement.

The rapidly increasing number of studies on the formulation of S-SEDDS shown in Table 1 demonstrates the rising interest in this type of products. Therefore, it is surprising that there are no S-SEDDS products yet in the market. The reasons could be related to the limited number of stability studies [61], and hence regulation issues, drug leakage and precipitation when diluted upon arrival in the GI fluids.

### 6.1. Examples of Instant Release S-SEDDS

Dixit and Nagarsenker [66] produced self-nanoemulsifying granules of ezetimibe using Type IV lipid formulation and they found a remarkable increase in dissolution of the drug compared to the non-formulated and further evaluation in vivo in rats showed a significant decrease in the total cholesterol levels compared to the control. Jannin et al. [25] also used Type IV lipid formulation and found that for each drug molecule (Piroxicam, Curcumin and Nifedipine), the system with the best performance during dispersion/digestion tests was not the same, as that which delivered the highest solvent capacity for the drug. However, Type IV surfactant-based formulation has been criticized for possible surfactant toxicity inducing irreversible changes in the GI membrane and for drug precipitation upon dilution in the GI fluids [62,106,107]. Accordingly, Type IV surfactant based formulations with a saturation level below 80% were suggested, in order to avoid drug precipitation during aqueous dispersion.

As it is shown in Table 1, most S-SEDDSs originate from Type III LFCS formulations using (a) mainly mono-, di- and triglycerides as oils and hydrophilic surfactants/co-surfactants or (b) combinations of hydrophilic with lipophilic surfactants and co-surfactants. Moreover, a number of lipophilic drugs with high LogP values (6.4 Lercanidipine, 5.9 Olmesartan medoxomil, 4.3 Sirolimus and 4.0 Ibuprofen) have been processed into S-SEDDS pellets from surfactant based Type IV LFCS using water dispersible surfactants [32,33,34,35], and a number of other lipophilic drugs (5.3 Repaglinide, 4.3 Sirolimus and 3.9 Celecoxib) have been processed into S-SEDDS using mixtures of hydrophilic with lipophilic surfactants [36,37,38].

Comparatively, from the three types of S-SEDDS presentations shown in Table 1, pellets and coarse powders form the majority with 41.2% and 38.2% respectively, while granules prepared using standard machinery form a smaller percentage 14.7% and powder processed into tablet form only a small percentage of 5.8%. Since tablets combine most of the desirable attributes of a solid dosage form, it is surprising that there are only few reported cases of tableted S-SEDDS [61]. Hence, the possibility of forming self-emulsifying pellets, powders and granules into tablets avoiding the loss of the self-emulsifying ability and the mechanisms involved, is a topic that merits further investigation.

Gao et al. [62] developed supersaturable self-emulsifying drug delivery systems of paclitaxel employing hydroxypropyl methylcellulose (HPMC-E5LV), as a precipitation inhibitor, in a conventional SEDDS formulation (Glyceryl dioleate, Cremophor EL, PEG 400). They found that this system was supersaturated with respect to paclitaxel and the supersaturated state was prolonged by the presence of 5% *w*/*w* HPMC in the formulation. In a simulated gastric fluid the S-SEDDS formulation yielded apparent solution concentrations, which were much higher than the equilibrium solubility of paclitaxel that was maintained for 2 h, suggesting that this formulation effectively produces and maintains a supersaturated drug solution in vitro. These in vitro findings were also visualized in in vivo studies where the SEDDS formulation provided a mean Cmax of only 13.1 ng/mL and an oral bioavailability of 0.9%, in comparison to the S-SEDDS with 5% *w*/*w* HPMC formulation which provided a 20-fold increase in Cmax (300 ng/mL) and an oral bioavailability of 9.5%.

In another study, Wei et al. [63] prepared S-SEDDS formulation of silybin (plant flavonoid) consisting of Labrafac CC, Cremophor RH40, Labrasol and 5% HPMC. Dilution of the S-SEDDS formulation in simulated gastric fluid resulted in a formation of a microemulsion with slow silybin precipitation, whereas rapid precipitation of silybin from S-SEDDS formulation without HPMC was noticed resulting in low drug concentration in the GI fluids. These in vitro results were also visualized in in vivo studies in rats, which indicated that the area under the concentration-time curve (AUC_0_→12 h) of the silybin in S-SEDDS increased by nearly 3-fold, in comparison to that obtained from SEDDS without HPMC.

### 6.2. Examples of Controlled Release S-SEDDS

Although the application of SEDDS is primarily intended for improvement of the absorption of poorly water soluble drugs, it would also be desirable to provide sustained release action in the case of low dose drugs with short biological half-life requiring frequent dosing. For this reason, the combinations of SEDDS with control release agents have been studied, in order to develop a matrix type controlled release S-SEDDS. As a result of this, matrix type spherical granules and pellets of S-SEDDS have been developed offering the benefits of both absorption improvement and sustained release [56,60].

Patil et al. [55] used colloidal silicon dioxide (CSD) in SEDDS formulations which served the dual purpose of reducing the amount of required solidifying excipients and aiding in slowing down the release of ketoprofen. Serratoni et al. [46] showed that it is possible to obtain control release of methyl and propyl paraben from pellets by first incorporating them into SEDDS (mono- and di- glycerides with Tween 80) to enhance the release rate. Following that, they applied the methods of extrusion/spheronization using MCC and lactose and finally by applying an HPMC/ethylcellulose polymeric water-insoluble coating, which contained a water soluble plasticizer and talc, they manage to control the release rate. Changing the coating thickness and/or pre-coating the pellets with a sub-coat of a water-soluble polymer can refine the control of the in vitro release of the drug and provide a range of release rate.

Agarwal et al. [50] investigated the effect of silica-based adsorbents with different chemical nature, specific surface area and particles sizes on the in vitro release behaviour of griseofulvin from SEDDS and S-SEDDS. Besides the enhanced dissolution rate of griseofulvin, as compared to the micronized drug powder, they also found that the SEDDS adsorbed onto magnesium aluminometasilicate (Neusilin^®^ UFL2, 5 μm) at 1:1 ratio, provided sustained drug release for a longer time. Setthacheewakul et al. [56] formulated tetrahydrocurcumin in self-emulsifying floating pellets by mixing SEDDS (Labrasol, Cremophor EL, Capryol 90, Labrafac PG) with adsorbent (colloidal silicon dioxide), sustained release and floating ability agent (glyceryl behenate) and disintegrants (pregelatinized starch and sodium starch glycolate). They used different weight proportions of glyceryl behenate and sodium starch glycolate with SEDDS and obtained pellet formulations with floating efficiency of 93% at 6 h and controlled release of tetrahydrocurcumin over an 8-h period, characterized by good stability for up to 6 months under intermediate and accelerated storage conditions.

Zhang et al. [60] developed sustained release self-emulsifying pellets of puerarin using Cremophor^®^ EL as the emulsifier, propylene glycol as co-emulsifier, various ratios of MCC and HPMC as pellet forming and sustained release agents respectively. They also prepared controlled release osmotic tablets of cyclosporine (CyA) by mixing SEDDS with adsorbents and osmotic tablet core excipients (sucrose, lactose monohydrate, polyethylene oxide, and partly pregelatinized starch) and subsequently transforming them into osmotic tablets. Nearly zero-order release was achieved [60]. Tao et al. [61] formulated sirolimus into self-emulsifying sustained release tablets by mixing SEDDS (Labrafil 1944CS Cremophor EL, Transcutol P with MCC and HPMC) and they also included an antioxidant in the formulation. US patent application US2012231083 [108] discloses a method for the preparation of sustained release cannabinoid medicament using SEDDS ingredients together with MCC and croscarmellose sodium to form an instant release layer or Μethocel Κ4Μ to form a slow release layer.

## 7. Conclusions

S-SEDDS present a promising strategy for developing instant or controlled release solid self-emulsifying formulations for low solubility and/or low permeability drugs, in order to improve their dissolution and absorption. Their manufacturability is good and straightforward and can be produced by employing conventional commonly available equipment, and thus require only minor changes in the followed production lines.

Reconstitution of submicron size emulsions from S-SEDDS in contact with water or GI fluids has been proven and absorption improvement has been verified by in vivo studies in animals. Colloidal silicon dioxide and silicate salts fulfil a double purpose: (a) they can use SEDDS emulsion as a binder, in order to form granules and pellets and (b) they consume large volumes of SEDDS emulsion for extrusion/spheronization; thus, increase the solidification capacity and the drug content in the dry product. Controlled release agents and crystallization inhibitors can be added to the pellet forming powder mixture to aid programming of drug release.

New hydrophobic surfactants of polyols esterified with fatty acids, which can be used in the SEDDS instead of oils and new water dispersible surfactants derived from glycerol esterified with ethoxylated fatty acids, have been found to contribute considerably to the increase of the solubilization capacity of the SEDDS—particularly for more lipophilic drugs. However, since powders, granules and pellets are not final dosage forms, processability into capsule or tablet dosage form is necessary and this topic needs further investigation to elucidate if there is any effect on the re-emulsification rate, the physical state of the drug in the formulation and on its dissolution and absorption.

Although the benefits of S-SEDDS for solubility and absorption improvement are clearly demonstrated and manufacturing equipment is readily available, they are not yet in the market as distinct dosage forms. To this aim it would help to collect data from long-term stability studies in order to establish the physicochemical stability of the S-SEDDS ingredients and ensure compatibility with the drug. This information together with data from pharmacokinetic studies on humans would encourage manufacturers to further invest for their development and the authorities to propose a regulation guide for approval.

## Figures and Tables

**Table 1:** List of drugs that have been formulated into solid self-emulsifying coarse powder, granules and pellets presented in chronological order.

Study	Drug/LogP/BCS Class	Oil	Surfactant/Cosurfactant	Powder Carriers	Presentation	Evaluation	Reference
1	Progesterone LogP = 3.87 Class IV	C8, C10 mono and di-glycerides (Imwitor 742^®^)	Tween 80	MCC	Pellets	In vitro dissolution & bioavailability improvement	Tuleu et al. 2004 [3]
2	Gentamicin LogP = n.a. Class III	PEG-8 caprylic capric glyceride (Labrasol)	Tween 80	Mg Aluminosilicate, silicon dioxide, calcium silicate	Powder filled into enteric capsules	In vitro dissolution & absorption enhancement	Ito et al. 2005 [12]
3	Nimesulide LogP = 2.60 Class II	C8, C10 mono and di-glycerides (Cithrol GMO^®^)	Tween 80	MCC, Lactose	Granules	In vitro dissolution & ex vivo permeability improvement	Franceschinis et al. 2005 [65]
4	Methyl Paraben LogP = 1.96 Propyl Paraben LogP = 3.04 Class n.c.	C8, C10 mono and di-glycerides (Imwitor 742)	Tween 80	MCC	Controlled release pellets	In vitro release enhancement	Serratoni et al. 2006 [46]
5	Diazepam LogP = 2.82 Class I	C18 mono and di-glycerides (Cithrol GMS)	Solutol HS 15	MCC	Pellets	In vitro dissolution & bioavailability improvement	Abdalla & Mader 2007 [11]
6	Ezetimibe LogP = 4.50 Class II	C8, C10 triglycerides (Miglyol, Labrafac lipophile WL 1349)	Capryol 90, Cremophor EL, Transcutol P	CSD	Coarse powders	In vitro dissolution improvement	Dixit & Nagarsenker 2008 [66]
7	Grizeofulvin LogP = 2.18 Class II	C8, C10 triglycerides (Captex 355)	Tween 80, Labrasol	Calcium silicate, Mg Aluminosilicate, silicon dioxide	Coarse powders	In vitro , dissolution improvement	Agarwal et al. 2009 [50]
8	Candesartan Cilexetil LogP = 4.0–5.1 Class II	C8, C10 triglycerides (Miglyol 812)	Tween 80, Labrasol	MCC, CSD, Sodium croscarmellose	Coarse powders	In vitro , dissolution & bioavailability improvement	Nekkanti et al. 2009 [67]
9	Nitrendipine LogP = 2.9 Class II	C8, C10 triglycerides (Miglyol 812)	Cremophor RH40, Tween 80, Transcutol P	MCC, Lactose, CSD, Crospovidone	Pellets	In vitro dissolution & absorption improvement	Wang et al. 2010 [68]
10	Tetrahydro-curcumin LogP = 3.5–4.0 Class IV	Propylene glycol dicaprylocaprate (Labrafac PG)	Capryol 90, Cremophor EL, Labrasol	MCC, CSD, Glyceryl behenate, Pregelatinised starch, Starch glycolate	Floating pellets—controlled release	In vitro solubility and dissolution improvement	Setthacheewakul et al. 2011 [56]
11	Piroxicam LogP = 3.0 Class II	Propylene glycol-monolaurate (Lauroglycol™ 90)	Cremophor EL, Transcutol HP	MCC, Lactose, PVP	Pellets	In vitro dissolution improvement	Franceschinis et al. 2011 [69]
12	Paliperidone LogP = 1.8 Class II	Oleic acid, C8, C10 mono and di-glycerides (Capmul MCM)	Tween 80	Mg Aluminometasilicate	Coarse powders	In vitro dissolution & ex vivo permeability improvement	Kanuganti et al. 2012 [51]
13	Sirolimus LogP = 4.3 Class II	n.a.	Labrafil 1944CS Cremophor EL, Transcutol P	MCC, Lactose, Na carboxymethyl starch	Pellets	In vitro dissolution & absorption improvement	Hu et al. 2012 [32]
14	Carbamazepine LogP = 2.45 Class II	C8, C10 triglycerides (Miglyol 812)	Tween 80, Cremophor RH 40	CSD, Mg Aluminometasilicate	Coarse powders	In vitro dissolution improvement	Milovic et al. 2012 [52]
15	Puerarin LogP = n.a. Class IV	Castor oil	Cremophor E4, Propylene glycol	MCC, HPMC	Pellets—sustained release	In vitro dissolution & bioavailability improvement	Zhang et al. 2012 [60]
16	Cilostazol LogP = 2.3 Class II	C8, C10 mono and di-glycerides (Capmul MCM)	Tween 80, Transcutol P	Mg Aluminometasilicate	Coarse powders	In vitro solubility improvement	Pund et al. 2013 [70]
17	Sirolimus LogP = 4.3 Class II	n.a.	Capryol, PGMC E-T PGS, glycofurol	Mannitol, Sucrose monopalmitate	Granules	In vitro solubility & dissolution improvement	Cho et al. 2013 [36]
18	Lercanidipine HCl LogP = 6.4 Class n.c.	n.a.	Gelucire 44/14, Labrasol, Transcutol P	Mg Aluminometasilicate	Coarse powders	In vitro , dissolution improvement	Kallakunta et al. 2013 [33]
19	Repaglinide LogP = 5.3 Class II	n.a.	Capryol 90, Cremophor EL, Solutol HS-15	MCC, Lactose, Kollidon CL, PVP	Pellets	In vitro , dissolution improvement	Desai & Negarsenker 2013 [37]
20	Ondasetron HCl LogP=2.40 Class II	Medium Chain Mono- and Diglycerides (Capmul MCM)	Labrasol, Tween 20	Silica, Mg Aluminometasilicate	Coarse powders	In vitro dissolution & bioavailability improvement	Beg et al. 2013 [53]
21	Bifendate LogP = 2.80 Class n.c.	Propylene Glycol Dicaprylate/Dicaprate (Miglyol^®^ 840)	Cremorphor^®^ EL, Solutol HS^®^ 15 (1:2, *w*/*w*)/Transcutol HP	MCC, lactose, mannitol	Pellets	In vitro dissolution & bioavailability improvement	Xiao et al. 2013 [47]
22	Atorvastatin calcium LogP = 5.7 Class II	Polyglycerol-3-oleate (Caprol 3GO)	Cremophor EL, Tween 20, Tween 80, *N*-methylpyrrolidone	MCC, CSD, Mg Aluminometasilicate	Coarse powders	In vitro dissolution & ex vivo permeability improvement	Agrawal et al. 2014 [50]
23	Olmesartan medoxomil LogP = 5.9 Class II	n.a.	Acconon Sorb-20, Tween 80, Carbitol	MCC, CSD, PVPP XL	Granules	In vitro dissolution & bioavailability improvement	Patel et al. 2014 [34]
24	Ibuprofen LogP = 3.97 Class II	n.a.	PEG 200 Labrasol	Mg Aluminometasilicate, MCC, Lactose	Pellets coated with SEDDS	In vitro , dissolution improvement	Krupa et al. 2014 [35]
25	Furosemide LogP = 2.03 Class II	C8, C10 triglycerides (Radia 7104)	Cremophor ELP, Cremophor RH40, Cremophor RH60	MCC	Pellets	In vitro , dissolution & solubility improvement	Nikolakakis et al. 2014 [71]
26	Propranolol LogP =3.48 Class II						
27	Oleanolic acid LogP na Class IV	Ethyl oleate	Labrasol, Transcutol P	Mannitol	Granules	In vitro , dissolution improvement	Ma et al. 2014 [27]
28	Simvastatin LogP = 4.68 Class II	Lauroglycol	Cremophor EL, Transcutol	MCC, Lactose, PVP	Granules	In vitro dissolution	Franceschinis et al. 2015 [72]
29	Glipiside LogP = 1.91 Class II	Phosphatidyl choline (Phosal 53 MCT), Capmul MCT	Tween 80, Transcutol	Silica (Syloid 244 FP)	Coarse powders	In vitro dissolution & bioavailability improvement	Agarwal et al. 2015 [50]
30	Celecoxib LogP = 3.9 Class II	n.a.	Capryol 90, Tween 20, Transcutol HP	CSD, Soluplus	Coarse powders	In vitro dissolution & bioavailability improvement	Chavan et al. 2015 [38]
31	Ibuprofen LogP = 3.97 Class II	C8, C10 triglycerides	Cremophor EL	MCC, CSD	Pellets	In vitro , dissolution improvement	Panagopoulou et al. 2015 [57]
32	Lercanidipine HCl Class n.c.	Rice brown oil/Clyceryl monooleate 1/9	Tween 80, Propionic acid	Mg Aluminometasilicate	Coarse powders	In vitro dissolution & absorption improvement	Suthar et al. 2016 [54]
33	Sirolimus LogP = 4.3 Class II	n.a.	Labrafil 1944CS Cremophor EL, Transcutol P	MCC, HPMC 100LV	Tablets—extended release	Stability improvement	Tao et al. 2016 [61]

Abbreviations: n.c.: Non classified; n.a.: Non applicable; MCC: Microcrystalline Celullose; CSD: Colloidal Silicon Dioxide; HPMC: Hydroxypropyl-methylcellulose; PVP: Polivinylpyrrolidone; BCS classification was taken from literature as follows: Progesterone, Tuleu et al. 2004 [3]; Gentamicin, Ito et al. 2005 [12]; Nimesulide, Mudie et al. 2012 [73]; Diazepam, Wu and Bennet 2005 [74]; Ezetimibe, Taupitz et al. 2013 [75]; Griseofulvin, Lindenberg et al. 2010 [76]; Candesartan Cilexetil, Nekkanti et al. 2009 [67]; Nitrendipine, Takano et al. 2006 [77]; Tetrahydrocurcumin, Wahlang et al. 2011 [78]; Piroxicam, Shohin et al. 2014 [79]; Paliperidone, Pandey et al. 2013 [80]; Sirolimus, Petruševska et al. 2013 [81]; Carbamazepine, Wu and Bennet 2005 [74]; Puerarin, Li et al. 2015 [82]; Cilostazol, Jinno et al. 2006 [83]; Lercaniditine, Non-classified; Repaglinide, Gao et al. 2013 [84]; Ondansetron, Beg et al. 2013 [53]; Atorvastatin calcium, Wu and Bennet 2005 [74]; Olmesartan medoxomil, Patel et al. 2014 [34]; Ibuprofen, Cristofoletti and Dressman 2017 [85]; Furosemide, Vogelpoel et al., 2010 [86]; Propranolol, Granero et al. 2010 [87]; Oleanolic acid, Tong et al. 2011 [88]; Simvastatin, Jiang et al. 2012 [60]; Glipizide, Zur et al. 2015 [89]; Celecoxib, Yazdanian et al. 2005 [90]; Lercanidipine, Suthar et al. 2016 [54]; LogP values were taken from ref. [91].

**Table 2:** Results of emulsion consumption, pellet diameter and shape of ibuprofen pellet batches prepared at different CSD/MCC ratios [57].

CSD/MCC	Consumption (mL)	% in Size Fraction (850–1200 μm)	Median Diameter (μm)	Aspect Ratio	Shape Factor (eR)
0/10	17	56.8	1070	1.101	0.433
3/7	25	74.4	1240	1.093	0.453
7/3	46	87.7	1250	1.130	0.419
10/0	58	79.1 ^#^	1310 ^#^	1.181	0.342

^#^ The higher median diameter for the CSD/MCC ratio 10/0 was due to a significant fraction of pellets >1200 μm.
